# Long-acting lipegfilgrastim and antimicrobials as vigorous primary prophylaxis in bendamustine-treated patients with indolent B cell non-Hodgkin lymphoma: a multicentric real-life experience

**DOI:** 10.1007/s00520-025-09743-9

**Published:** 2025-07-14

**Authors:** C. Giordano, M. Picardi, A. Vincenzi, A. Scarpa, A. Lombardi, M. Carchia, V. Damiano, R. Bianco, F. Trastulli, F. Ronconi, M. Annunziata, F. Pane

**Affiliations:** 1 Department of Clinical Medicine and Surgery, Federico II University Medical School, Via Sergio Pansini, 5, Naples, 80131 Italy; 2 Department of Public Health, Federico II University Medical School Naples, Via Sergio Pansini, 5, Naples, 80131 Italy; 3 Hematology Unit, Antonio Cardarelli Hospital of National Importance, Naples, Italy

**Keywords:** Febrile neutropenia, B cell non-Hodgkin lymphomas, Lipegfilgrastim, Primary prophylaxis

## Abstract

Febrile neutropenia (FN) is one of the most important clinical signs of infection, especially for older patients with indolent B cell non-Hodgkin lymphomas (iBC-NHLs) receiving frontline immune-chemotherapy with bendamustine-rituximab (BR). Data on the optimal strategy for infection prophylaxis from the start of chemotherapy until 1 month after the last cycle is scanty in this setting of patients, and for this reason, we carried out a multicentric retrospective study on vigorous primary anti-infectious prophylaxis consisting of lipegfilgrastim, trimethoprim-sulfamethoxazole, and acyclovir. From January 2017 to January 2022, 200 patients met the inclusion criteria and were enrolled in the final analysis. As per the primary endpoint, during the immune-chemotherapy period, the overall incidence of FN was 6% consisting of fever of unknown origin (2%), clinically documented infections (2.5%), and microbiologically documented infections (1.5%). Chemotherapy disruption for a delay of at least 1 week related to FN that required hospitalization was recorded in 1% of patients (*n* = 2). Prophylaxis was well tolerated with grade 3 toxicity (bone pain) in only 10% of patients and was successfully managed with paracetamol or tramadol. Systematic, prompt, and sustained use of vigorous primary anti-infectious prophylaxis was able to reduce the rate of fever episodes, thus averting parenteral antimicrobial administrations, hospitalizations, and immune-chemotherapy disruption.

## Introduction

Real-world experience in clinical practice shows a gradual transition from multiagent chemo-immunotherapy (rituximab, cyclophosphamide, doxorubicin, oncovin, prednisone [R-CHOP]) to bendamustine plus rituximab (BR) for front-line treatment of indolent B cell non-Hodgkin lymphomas (iBC-NHL) [1]. However, increased incidence of opportunistic infections following bendamustine exposure has raised questions about safety and widespread adoption, especially for older patients [2]. Febrile neutropenia (FN) is one of the most important clinical signs of infection during chemotherapy treatment and is characterized by an absolute neutrophil count (ANC) < 1000/mm^3^ and at least one temperature measuring of ≥ 38 °C [3]. The American Society of Clinical Oncology and the National Comprehensive Cancer Network recommend the use of granulocyte colony-stimulating factors (G-CSFs), which have been developed to stimulate the proliferation and differentiation of neutrophils in patients receiving cytotoxic agents [3]. The routine use of G-CSFs from the first cycle of myelosuppressive chemotherapy, i.e., primary prophylaxis, is indicated when the overall FN risk is greater than 20%. If the risk is lower than 20%, a secondary prophylaxis is suggested, consisting of post-chemotherapy G-CSF administration for patients who experienced a neutropenic complication from a previous cycle and where a dose reduction would compromise disease-free or overall survival, or treatment outcome. For managing cancer patients undergoing chemotherapy during the COVID-19 pandemic, the European Society for Medical Oncology multidisciplinary expert consensus states expanding routine indication of G-CSF for patients with intermediate (10%–20%) risk of FN and specifically for older patients with comorbidities [4].

An important issue here is also the type of G-CSF to employ: filgrastim is a non-pegylated short-acting form of G-CSF, used at the daily dose of 5 μg/kg, until the end of neutropenia, according to the myelosuppressive grade of chemotherapy schedules; pegfilgrastim is a pegylated long-acting recombinant form of G-CSF with extended half-life used to decrease the incidence of infections in patients with non-myeloid malignancies, receiving myelosuppressive chemotherapy [3] and requiring less frequent administrations (single dose administration per chemotherapy cycle) than non-pegylated G-CSF. Lipegfilgrastim is a novel glyco-pegylated long-acting G-CSF that was approved by the European Medicines Agency in 2013 to prevent chemotherapy related neutropenia with a once-per-cycle administration. A phase III study on the efficacy and safety of lipegfilgrastim versus pegfilgrastim in patients with breast cancer receiving doxorubicin/docetaxel chemotherapy [5] investigated the pharmacokinetics profile and demonstrated that the area-under-the-curve parameters were almost 50% higher for lipegfilgrastim compared with pegfilgrastim. The activity of a 6-mg lipegfilgrastim dose was expected to be greater than that of pegfilgrastim resulting in a trend towards a higher effect for lipegfilgrastim in terms of anti-neutropenic activity [5]. Moreover, in Bond et al. meta-analysis [6] on the indirect treatment comparison of lipegfilgrastim with pegfilgrastim and filgrastim for the reduction of chemotherapy-induced neutropenia-related events, lipegfilgrastim had a statistically significantly lower absolute neutrophil count recovery time.

In addition, data on the optimal strategy for antimicrobial prophylaxis regarding acyclovir and trimethoprim-sulfamethoxazole efficacy or the proper administration schedule is scanty in the setting of hematological patients undergoing immuno-chemotherapy. An antiviral prophylaxis with acyclovir has for long been the mainstay for prophylaxis of herpes virus (HSV) and varicella zoster virus (VZV) in this setting of patients; a systematic review of the Multinational Association of Supportive Care in Cancer (MASCC)/International Society of Oral Oncology (ISOO) showed that acyclovir is effective in preventing herpetic viral disease at 400 mg BID dose [7]. Moreover, trimethoprim-sulfamethoxazole has always been of particular interest for prophylactic treatment as it presents a wide range of action against common bacteria, parasites including toxoplasmosis and fungi and yeasts such as *Pneumocytis jiroveci*. For this reason, together with acyclovir, it is recommended for immunocompromised patients, especially in an older lymphoma patient setting or when BR is employed [8, 9]. Nonetheless, a standardized prophylactic regimen has never been established for this type of patient, especially regarding the type of anti-infective agents and timing schedule.

In keeping with proper prophylaxis management, data on the optimal G-CSF strategy, i.e., primary vs. secondary prophylaxis and/or short-acting vs. long-acting form, as well as the optimal antimicrobial strategy against pneumocystis and herpes virus, is scarce in the setting of patients undergoing front-line treatment with BR for iBC-NHL [2].

Thus, we carried out a multicentric retrospective study in iBC-NHL receiving a primary anti-infection prophylaxis when receiving BR. We defined an intensified primary antimicrobial prophylaxis (IPAMP) consisting in a systematic, prompt, and sustained use of three antimicrobial/prophylactic agents: lipegfilgrastim, trimethoprim-sulfamethoxazole and acyclovir. Herein, we report on the mature results of this study.

## Material and methods

### Study design

This was a retrospective study using the medical records and local database of the Hematology Unit of the Federico II University of Naples (Italy), Oncology Unit of the Federico II University of Naples (Italy), and Hematology Unit of the Antonio Cardarelli Hospital of national importance of Naples (Italy). All necessary approvals were obtained from the Ethic Committee of the Federico II University of Naples (approval protocol number: 66/2024). The acquisition of a written informed consent was obtained from each patient undergoing immuno-chemotherapy. The form contained detailed information regarding treatment risk.

These units had similar internal guidelines for the management of patients with lymphoma and the same local ethics committee.

From January 2017 to January 2022, consecutive untreated iBC-NHLs patients, with clinical indication to receive six 4-week cycles of rituximab (R) (375 mg/m^2^ *i.v.* on day 1) plus bendamustine (90 mg/m^2^ *i.v.* days 1–2) according to the standard schedule [10], undergoing IPAMP, were enrolled. IPAMP was used as routine practice for iBC-NHLs undergoing BR treatment in the three clinical units.

### Inclusion and exclusion criteria

Patients with a histological diagnosis of iBC-NHLs [11], age ≥ 18 years, and WHO performance score 0–2 were screened for enrollment. Eligible criteria were features requiring starting immuno-chemotherapy treatment, Ann Arbor stage III or IV plus signs of impaired hematopoiesis (hemoglobin < 10 g/dL, ANC < 1500/mm^3^, or platelet count < 100 × 10^9^/L), presence of B symptoms, large tumor burden (three areas > 5 cm, or one area > 7.5 cm), bulky disease with impingement on internal organs, progressive disease (defined as a more than 50% increase of tumor mass within 6 months), symptomatic splenic enlargement or palpable at 6 cm from costal arch, and/or hyperviscosity syndrome [12]. The exclusion criteria were patients receiving a treatment regimen other than BR, a histological diagnosis of aggressive lymphoma, a concomitantly neoplastic disease, or autoimmune disease (for which preexisting immunosuppressant therapy was employed).

### Data collection

Data collection forms were sent to the data managers of the three centers requesting the following information: patient demographics, European Cooperative Oncology Group performance status (ECOG), histological diagnosis, past medical history, radiological assessment with fluoro-desossy-glucose positron emission computed tomography (FDG-PET/TC) before BR scheme employment and at end-of-treatment (EOT), bone marrow results, current lymphoma immuno-chemotherapy induction treatment, dose reductions and delays, prophylactic antimicrobial and supportive medication, and blood results before induction therapy. Data forms included full blood count, serum biochemistry, immunoglobulin values, grade 3 to 5 infections, opportunistic infections, impact of adverse events on dose reductions, delays and treatment discontinuation, and deaths related to bendamustine.

Data regarding the duration of severe neutropenia in each cycle, the lowest ANC level reached (ANC nadir) in each treatment cycle, and time to ANC nadir and to recovery (defined as a return of ANC to ≥ 2.000/mm^3^) were also retrieved.

### Intensified primary anti-microbial prophylaxis regimen

**Fig. 1:**
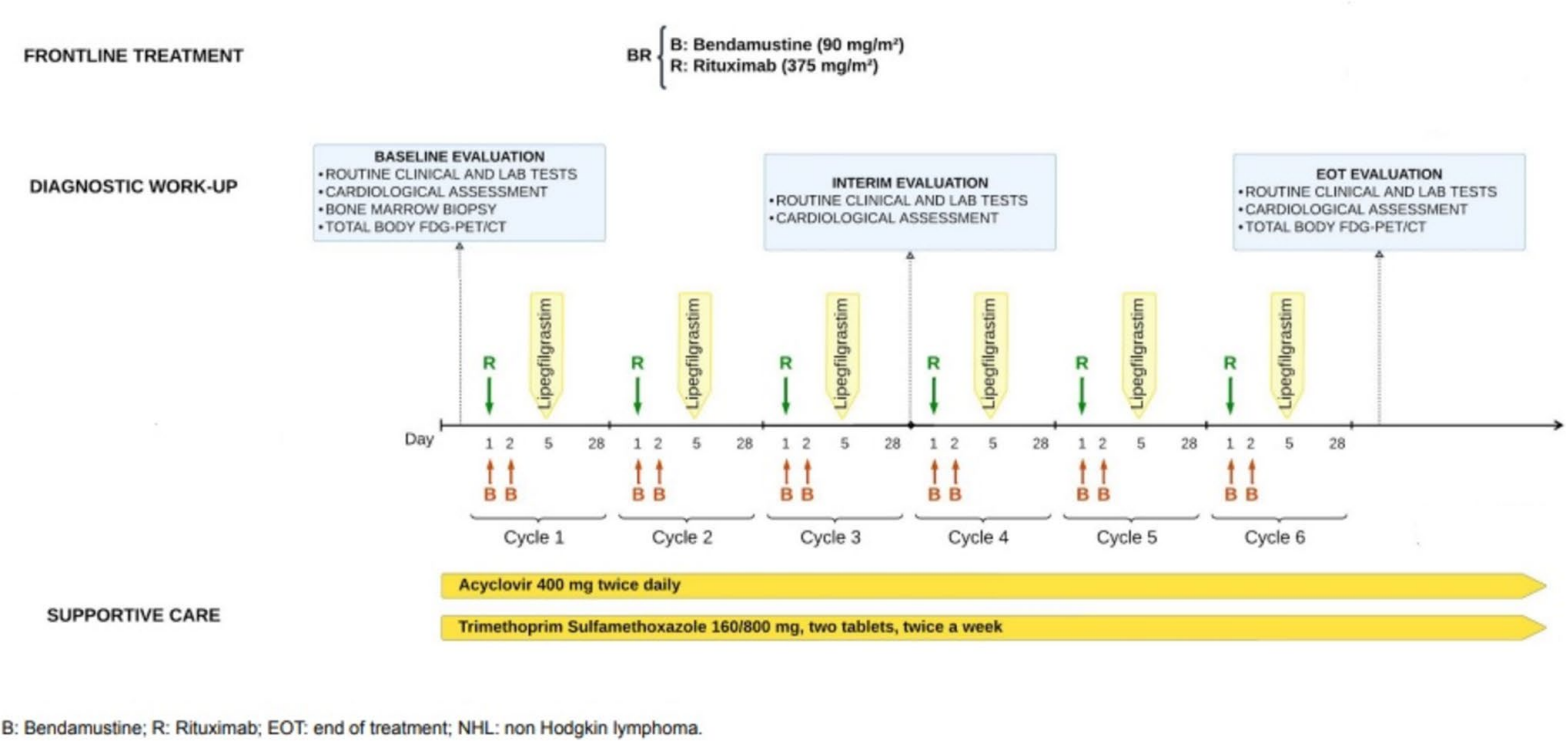
Intensified primary anti-microbial prophylaxis regimen for patients undergoing BR immunochemotherapy. Lipegfilgrastim 6 mg was administered subcutaneously on day 5 of each BR cycle (from the first until the last 4-week cycle), trimethoprim-sulfamethoxazole at 960 (160 + 800) mg orally every 12 h for 2 days a week, and acyclovir at 800 mg orally daily from 1 week prior to the start of chemotherapy until one month after the last cycle

Primary antimicrobial prophylaxis drugs were routinely administered for each patient, constituting the IPAMP schedule, as follows: lipegfilgrastim (a glycopegylated modification of pegfilgrastim): 6 mg was administered subcutaneously on day 5 of each BR cycle (from the first until the last 4-week cycle), trimethoprim-sulfamethoxazole at 960 (160 + 800) mg orally every 12 h for 2 days a week and acyclovir at 800 mg orally daily from 1 week prior to the start of chemotherapy until 1 month after the last cycle (Fig. 1). According to the enrolled center prophylaxis guidelines, for every 4-week cycle, all patients routinely received methylprednisolone at 200 mg i.v. and diphenhydramine at 50 mg i.v. on days 1 and 2, febuxostat at 80 mg orally on days 1 to 5 (plus hyperhydration).

### Study endpoints

The primary endpoint was FN incidence during the induction immune-chemotherapy period, secondary endpoints were FN-related chemotherapy disruption (regarding dose-dense and/or dose-intensity of the schedule), days of hospitalization due to FN and prophylaxis-related side effects (grade ≥ 3 Common Terminology Criteria for Adverse Events version 5.0 [CTCAE]) [13].

In regards to the secondary endpoint of FN-related chemotherapy disruption, if the leukocyte count was less than 2000/mm^3^ prior to a scheduled cycle, the treatment cycle was delayed for at least 1 week and thus a *time-disruption* was recorded; while, for bendamustine dose reduction to 70 mg/m^2^ when leukocyte count was less than 1000/mm^3^ on two consecutive days between cycles, a *dose-disruption* was recorded.

### Infection diagnostic work-up

During the study period, as part of the three clinical units’ institutional guidelines for post-chemotherapy supportive care, in patients with FN, i.e., an ANC < 1000/mm^3^ and at least one temperature measuring of ≥ 38 °C, a basic infection diagnostic work-up was performed. Two or more sets of blood cultures at fever onset and at 24–48 h for bloodstream isolate identification and antimicrobial susceptibility testing were performed; urine test analysis, liver and renal function tests, nasopharyngeal swab, and sputum sample culture test were also part of the basic diagnostic work-up. If the fever persisted for over 72 h, the diagnostic work up was widened including serum cytomegalovirus-DNA test screening, evaluation of serum galactomannan and beta-d-glucan, chest radiography or thorax computed tomography (CT) scans including bronchoscopy and bronchoalveolar lavage (if signs of pulmonary infection were detected), stool sample culture test, and ultrasound abdomen evaluation.

### Adverse events

Safety was assessed by the incidence of all side effects related to the IPAMP schedule and were reported according to the National Cancer Institute CTCAE v.5.0 [13]. Biochemical tests were routinely performed, and changes in clinical chemistry, especially for renal function, were recorded over time. Physical examination was also routinely performed, and particular attention was paid to skin evaluation regarding possible erythematous reactions related to the IPAMP schedule.

In addition, AEs were classified as “bone-pain–related symptoms” with a comprehensive definition including the AE terms arthralgia, back pain, bone pain, neck pain, myalgia, and other musculoskeletal symptoms.

### Statistical analysis

Patient and disease characteristics, FN rates, toxicity, and safety data were reported descriptively as number and percentage, or as median and range. For statistical analysis, the evaluated factors included age stratification (years: < 70 vs. 70–79 vs. ≥ 80), B symptoms, large nodal masses (LNMs), nodal and/or extra-nodal sites involved, stage IV, and international prognostic score (IPS) risk group at baseline.

Continuous data were expressed as mean and standard deviation (SD) or median and. interquartile range (IQR) depending on whether they presented a normal distribution, and. between-group comparisons were performed using student T-tests or Wilcoxon’s rank. tests, depending on the distribution of the variable. Categorical data were expressed as. frequencies and associated percentages. Values of *p* < 0.05 were considered statistically significant. Statistical analysis was conducted using SPSS software IBM NY (version 26.0).

## Results

### Patient characteristics

From January 2017 to January 2022, 200 untreated iBC-NHL receiving IPAMP for front-line BR met the inclusion criteria and were enrolled in the final analysis. Overall, the median age was 66 years (range, 40–82 years) and 53% were male. Histological iBC-NHL subtypes were follicular lymphoma in the 59.5%, marginal zone lymphoma (25.5%), mantle cell lymphoma (10.5%), small lymphocytic lymphoma (3%), and lymphoplasmacytic lymphoma (1.5%). The 72% of the patients enrolled had stage IV according to the Ann Arbor classification and 31% presented B symptoms (Table 1). All patients systematically received IPAMP treatment as scheduled except for three cases (1.5%) that were intolerant to trimethoprim-sulfamethoxazole and received 500 mg erythromycin per day for one week per each BR cycle.

**Table 1:** Patients’ characteristics

Number of patients	200
Sex (female/male)	109/91 (53/47%)
Age	
Median	66
Range	40–82
Type of disease	
Follicular lymphoma	119 (59.5%)
Marginal zone lymphoma	51 (25.5%)
Mantle cell lymphoma	21 (10.5%)
Small lymphocytic lymphoma	6 (3%)
Lymphoplasmacytic lymphoma	3 (1.5%)
Stage	
II	11 (5%)
III	46 (23%)
IV	145 (72%)
B symptoms	63 (31.5%)
Bulky disease	90 (45%)
Bone marrow involved	77 (38.5%)
Extra-nodal involved sites ≥ 1	60 (30%)
LDH > 240 U/L	156 (78%)

Values are *n* (%) unless otherwise specified

Stage is reported according to the Ann Arbor staging system: stage I indicates that the lymph node is located in a single region, usually one lymph node and the surrounding area. Stage II indicates that lymph node is located in two separate regions, an affected lymph node or lymphatic organ and a second affected area, and that both affected areas are confined to one side of the diaphragm—that is, both are above the diaphragm, or both are below the diaphragm. Stage III indicates that it has spread to both sides of the diaphragm, including one organ or area near the lymph nodes or the spleen. Stage IV indicates diffuse or disseminated involvement of one or more extralymphatic organs, including any involvement of the liver, bone marrow, or nodular involvement of the lungs

Histological diagnosis is reported according to WHO 2022 criteria^11^

### Primary endpoint

Overall, five patients recorded a total of 16 neutropenic episodes (8%). The mean of nadir of ANC was 1554/mm^3^ (range, 800–1920/mm^3^) for a mean duration of 4 days (range, 2–8 days) until recovery. Most of the neutropenic episodes (60%, *n* = 10/16) were recorded in the 5th and 6th cycle of BR, while the 40% were equally recorded in the 2nd, 3rd and 4th cycle. No neutropenic episodes were recorded in the 1 st cycle of BR.

The incidence of FN during the chemotherapy period was 6%: 4 patients (2%) had FN of unknown origin, 5 patients (2.5%) had FN with clinically documented infection episodes, and 1.5% of patients had a microbiologically documented infection episode (Table 2). Regarding the FN of unknown origin, 2 patients recorded the episode at the 2nd cycle while the other 2 at the 5th and 6th cycle of BR, respectively. For FN with clinically documented infection: 3 patients had an infection of the respiratory tract (2 with radiological signs of pneumonia) recorded at the 3rd cycle of BR for 2 patients and at the 5th cycle for one patient; two had a clinically documented infection of the gastrointestinal tract (4th and 5th cycle of BR, respectively). Regarding the microbiologically documented infection episodes: 2 patients had a bronchoalveolar lavage (BAL) positive for *Staphylococcus* spp. and *Pseudomonas* spp. and 1 patient a serum cytomegalovirus DNA positivity, all recorded at the 5th cycle of BR. All of them had clinical and radiological findings of pneumonitis.

**Table 2:** Characteristics of neutropenic episodes

Neutropenic episodes without infectious symptoms	16 (8%)
FN episodes*	12 (6%)
FN of unknown origin	4 (2%)
FN with clinically documented infection	5 (2.5%)
Site/source of infection	
*Upper respiratory tract*	1 (0.5%)
*Lower respiratory tract*	2 (1%)
*Gastrointestinal tract*	2 (1%)
with radiological signs of infection	2 (1%)
FN with microbiologically documented infections	3 (1.5%)
BAL positivity	2 (1%)
*Staphylococcus* spp.	1 (0.5%)
*Pseudomonas* spp.	1 (0.5%)
CMV-DNA positivity	1 (0.5%)
Hospitalization days, median (range)	7 (2–10)

Values are *n* (%) unless otherwise specified

^*^Each episode is recorded for single patient

*FN*, febrile neutropenia; *BAL*, bronchoalveolar lavage; *CMV*, cytomegalovirus

Radiological signs: chest radiography or CT scans suspected of pneumonia

### Secondary endpoints

Chemotherapy disruption related to FN was recorded in 1% of patients (*n* = 2) (*time disruption*), while *dose-disruption* was not recorded for the entire population. Time disruption was recorded for a delay in treatment cycle of 7 and 15 days, respectively.

Two patients were hospitalized due to FN (and consequently disrupted the chemotherapy regimen) for a median period of 7 days (2–10); in particular, both patients were diagnosed with pneumonia (Table 2) and required intravenous antibiotic treatment and oxygen therapy but were dismissed at home with full recovery.

Prophylaxis-related side effects were recorded according to CTCAE v.5.0 toxicity criteria [13]. Prophylaxis was well tolerated with grade 3 toxicity (bone pain related symptoms) in only 10% of patients (5% were < 70 years old, 3% were in the age group 70–79 years, and 2% were older than 80 years) and was successfully managed with paracetamol or tramadol (in 5 patients refractory to paracetamol). Regarding grade 1–2 bone pain, toxicity was recorded in 35% of patients, while grade 1–2 headache was recorded in 20% of patients.

### Statistical analysis results

No significant statistical differences were found among the evaluated factors of B symptoms, LNMs, stage, and international prognostic score (IPS) risk group at baseline for both primary and secondary endpoint except for age stratification (years: < 70 vs. 70–79 vs. ≥ 80) for which neutropenic episodes were more frequently recorded in patients older than 70 years vs. < 70 years (χ2 test, *P* = 0.007).

## Discussion

In literature, several published studies, such as Rapoport et al. [14], Aapro et al. [15], and Zheng et al. [16], exploit the topic on prevention of fever for patients with cancer (including NHLs) and intermediate risk of FN. Although the authors underscore the need for interventions that could mitigate the infectious toxicity, there were no unequivocal conclusions regarding prophylactic G-CSF and use of antimicrobials.

In patients with iBC-NHL receiving front-line treatment with BR, an intensified primary prophylaxis with lipegfilgrastim, acyclovir, and trimethoprim-sulfamethoxazole was evaluated in terms of FN episodes possibly causing chemotherapy regimen disruption and hospitalization. In our retrospective series, a total of 6% of FN episodes were recorded with FN with an unknown origin rate of 2%, FN with clinically documented infection rate of 2.5%, and with microbiologically documented infections of 1.5%. Chemotherapy disruption was recorded in 1% of patients due to hospitalization for FN and pneumonia.

Our study focused on specific intervention against infections based on lipegfilgrastim (a glycopegylated modification of filgrastim); compared with pegfilgrastim, lipegfilgrastim has been reported to have a statistically significantly lower absolute neutrophil count recovery time [6].

In *Supportive Care in Cancer* of 2017 [17], we have already reported encouraging single-center safety results of a prospective trial in 61 younger adult patients receiving first-line BR and pegfilgrastim, acyclovir and trimethoprim-sulfamethoxazole prophylaxis for iBC-NHL during the 2014–2016 period. Infectious incidence was evaluated during the immunochemotherapy regimen duration. Overall, pegfilgrastim-based prophylaxis regimen was associated with a rate of FN of 18% (11/61 patients), and chemotherapy disruption occurred in 3% of patients. Pegfilgrastim-related extrahematological toxicity (bone pain) of grade ≥ 3 was observed in 8/61 patients (13%), managed successfully with paracetamol, except for one patient who had to stop pegfilgrastim treatment. According to our recent data, lipegfilgrastim-based prophylaxis was characterized with a lower rate of FN (6% vs. 18%, *P* = 0.039) and was well tolerated (grade ≥ 3 bone pain, 10%), and all patients received the expected total number of lipegfilgrastim injections per immunochemotherapy regimen. However, our current study presents several limitations: its retrospective nature, the absence of clinical follow-up data, and the single cohort study design. For these reasons, our findings need to be validated in prospective studies with long-term follow-up to provide a more comprehensive evaluation of the impact of the IPAMP prophylaxis regimen on the infection rate, which needs to be considered due to the high risk associated with late lymphopenia from bendamustine and the possible maintenance treatment of rituximab every 2 months for 2 years.

Compared with the data of Rapoport et al. [14], the number needed to treat (NNT) with the IPAMP scheme to prevent 1 episode of FN was 14. This was a post hoc comparison between two different prophylaxis approaches against infections in different patient populations (several underlying malignant diseases in Rapoport et al. [14] vs. iBC-NHL in Giordano et al. [present study]). In our opinion, the NNT of 14 should be considered clinically effective [18] since in each patient the dose-dense/dose-intensity of the treatment schedule is crucial for the outcome of the underlying cancer [14–17], and FN occurrence is usually the first cause of chemotherapy disruption.

In conclusion, we defined a vigorous primary anti-infectious prophylaxis (consisting of lipegfilgrastim, trimethoprim-sulfamethoxazole, and acyclovir), whose systematic, prompt, and sustained use was able to reduce the rate of fever episodes thus declining parenteral antimicrobial administration and hospitalizations required for febrile neutropenia or other infectious complications in patients with iBC-NHL receiving front-line BR regimen.

## Data Availability

No datasets were generated or analysed during the current study.
